# *In Vivo* Detection of Low Molecular Weight Platform Chemicals and Environmental Contaminants by Genetically Encoded Biosensors

**DOI:** 10.1021/acsomega.3c01741

**Published:** 2023-06-23

**Authors:** Thomas Bayer, Luise Hänel, Jana Husarcikova, Andreas Kunzendorf, Uwe T. Bornscheuer

**Affiliations:** Department of Biotechnology and Enzyme Catalysis, Institute of Biochemistry, University of Greifswald, Felix-Hausdorff-Strasse 4, 17487 Greifswald, Germany

## Abstract

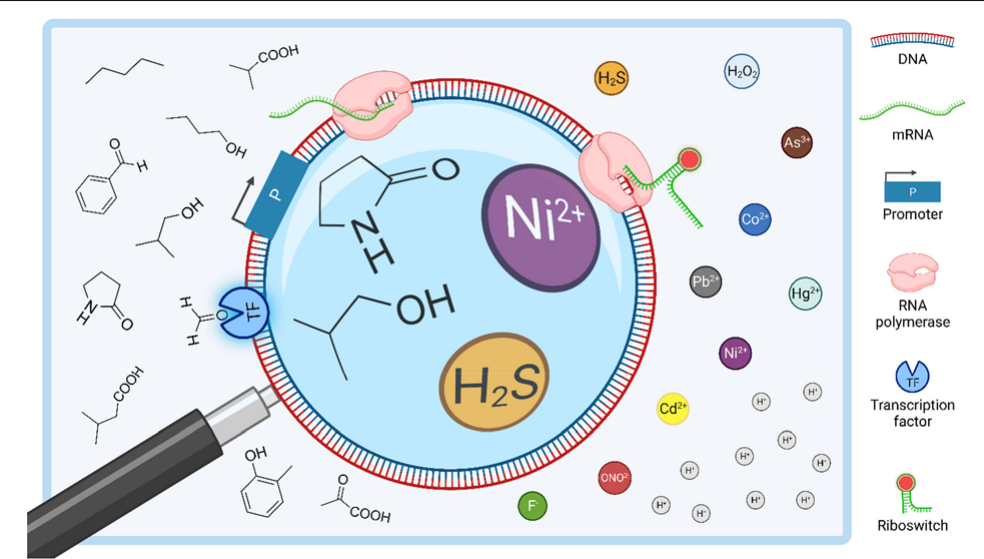

Genetically encoded biosensor systems operating in living cells are versatile, cheap, and transferable tools for the detection and quantification of a broad range of small molecules. This review presents state-of-the-art biosensor designs and assemblies, featuring transcription factor-, riboswitch-, and enzyme-coupled devices, highly engineered fluorescent probes, and emerging two-component systems. Importantly, (bioinformatic-assisted) strategies to resolve contextual issues, which cause biosensors to miss performance criteria *in vivo*, are highlighted. The optimized biosensing circuits can be used to monitor chemicals of low molecular mass (<200 g mol^–1^) and physicochemical properties that challenge conventional chromatographical methods with high sensitivity. Examples herein include but are not limited to formaldehyde, formate, and pyruvate as immediate products from (synthetic) pathways for the fixation of carbon dioxide (CO_2_), industrially important derivatives like small- and medium-chain fatty acids and biofuels, as well as environmental toxins such as heavy metals or reactive oxygen and nitrogen species. Lastly, this review showcases biosensors capable of assessing the biosynthesis of platform chemicals from renewable resources, the enzymatic degradation of plastic waste, or the bioadsorption of highly toxic chemicals from the environment. These applications offer new biosensor-based manufacturing, recycling, and remediation strategies to tackle current and future environmental and socioeconomic challenges including the wastage of fossil fuels, the emission of greenhouse gases like CO_2_, and the pollution imposed on ecosystems and human health.

## Introduction

Today’s environmental and socioeconomic challenges are multifaceted. Main contributors are the pollution of water, air, and soil, as well as the production of waste, paired with insufficient disposal and recycling strategies. Equally problematic are the depletion of natural resources and a strong dependence on fossil fuels. The latter lead to the ever-rising emission of greenhouse gases such as the single-carbon (C_1_) compounds carbon dioxide (CO_2_) or methane (CH_4_).^1,2^ To overcome these challenges, robust and efficient tools for the detection and quantification of contaminants as well as unprecedented strategies for the utilization of C_1_ molecules are highly desired. C_1_ compounds are considered important carbon sources in the future to both sustainably produce value-added chemicals and generate energy. Additionally, improved recycling and remediation schemes are vital to achieving closed-loop economies and reducing, ideally preventing, the pollution imposed on ecosystems and human health.^1,3,4^

Complementary to recent reviews highlighting advancements in natural and artificial carbon fixation pathways,^1,5,6^ this review will focus on genetically encoded biosensor systems operating in living cells, suitable to detect immediate fixation products (e.g., formate and pyruvate) and their industrially relevant derivatives. Physicochemical biosensor designs employed *in vitro* were recently reviewed by others and are not included herein.^7,8^ Furthermore, this condensed review will feature devices for the biosensing of (inorganic) contaminants that directly relate to anthropogenic activities, including, but not limited to, heavy metal (HM) and fluoride (F^–^) ions, as well as chalcogen-containing compounds such as hydrogen sulfide (H_2_S) and hydrogen peroxide (H_2_O_2_). Many of these chemical entities, organic and inorganic, have low molecular masses (<200 g mol^–1^) and can be difficult to analyze due to their physicochemical properties. Hence, analysis regularly requires laborious sample preparation and specialized instruments for detection and quantification.^2^

**Figure 1:**
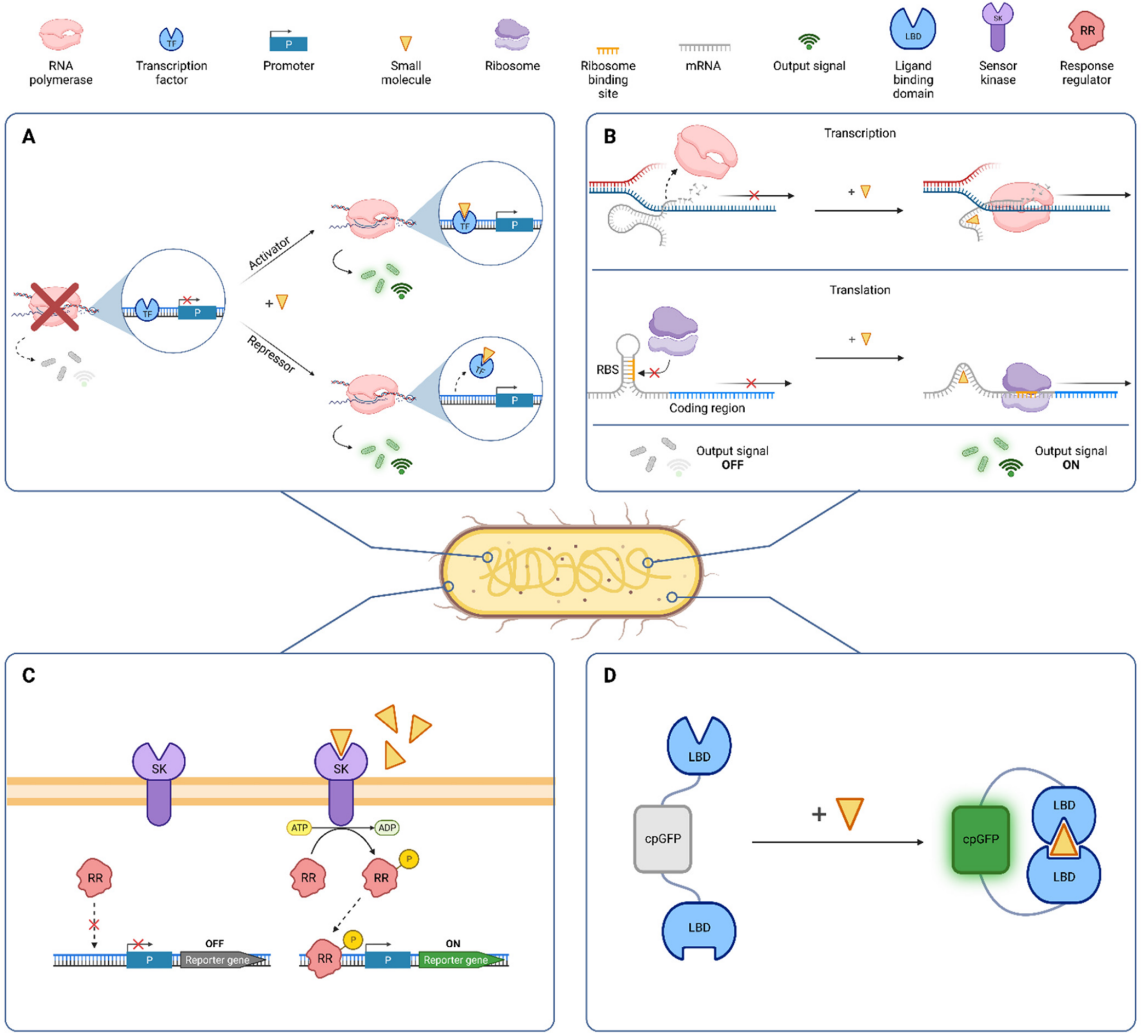
Types of genetically encoded biosensor systems. (A) (Allosteric) transcription factors (TFs) can act as activators or repressors. Whereas in the absence of the target small molecule no readable output signal is generated (OFF state), ligand binding to the TF facilitates promoter recognition or promoter clearance, respectively, resulting in the reporter gene transcription (ON state). (B) Riboswitches (RSWs) act on the co- or posttranscriptional level. Regulatory mechanisms involve the formation of hairpin terminators in the absence of the ligand, leading to truncated transcripts, or the sequestration of the RBS, impeding translation (OFF state). Binding of the small molecule, triggers a conformational change in the mRNA (mRNA), enabling transcription and translation, respectively (ON state). (C) Two component systems (TCS) consist of a membrane-bound sensor kinase. Ligand binding outside of the cell activates the kinase domain, subsequently phosphorylating a response regulator at the expense of adenosine triphosphate (ATP). The activated regulator protein recognizes its cognate promoter, enabling reporter gene expression (ON state). (D) Fluorescent probes such as variants of circularly permuted green fluorescent protein (cpGFP) act as sensors on the post-translational level. In cpGFPs, the termini are fused to sensing domains and fluorescent intensity is modulated in the presence of target small molecules, generating the output signal. Additionally, enzyme-based biosensor systems can directly generate output signals by converting target substrates (not shown; see main text). Figure created with BioRender.com.

The implementation of genetically encoded biosensor systems offers solutions to these obstacles. Generally, biosensors consist of two functionally linked components: a sensing and a transduction module.^9^ Transcription factors (TFs; Figure 1A) and riboswitches (RSWs; Figure 1B) can be used as sensory parts to detect the presence of a chemical entity (i.e., the input signal). Minimal TFs consist of a DNA-binding domain (DBD) and a ligand-binding domain (LBD), while RSWs comprise oligonucleotides with a length of 30–80 nucleobases, so-called RNA aptamers that specifically bind a target small molecule. Ligand binding regularly triggers a conformational change in TFs and RSWs, regulating the expression of reporter or pathway genes, which act as transducers. Transducers decode the input into a readable output signal (e.g., fluorescence, luminescence) encoded by different reporter genes (Table 1).^9−11^ Complementary, two-component systems (TCS) have been introduced only recently as a class of biosensors. TCS consist of a membrane-bound sensor kinase that binds a small molecule in the environment. This input signal results in the activation of the intracellular kinase domain, phosphorylating a response regulator. The activated regulator can bind to its cognate promoter sequence, ultimately, driving reporter gene expression (Figure 1C).^12^ Lastly, fluorescent probes (Figure 1D) or certain enzymes such as luciferases (Table 1) directly generate readable outputs in the presence of target small molecules and will also be featured in this review.

**Table 1:** Featured Biosensor Systems

sensor	input (ligand)	output (reporter)	host	operational range	ref
AlkR (TF)	*n*-alkanes (≥C_12_)	GFP (fluorescence)	*A. baylyi*	1	(37)
AlkS (wildtype TF)	*n*-alkanes (C_5_–C_10_), primary SMCAHs (≥C_5_)	GFP (fluorescence)	*E. coli*	0.5–10 mM^2^	(34)
AlkS (mutant TF)	*n*-alkanes (C_5_–C_9_), various SMCAHs (C_3_–C_5_)	GFP (fluorescence)	*E. coli*	1–100 mM^3^	(34 and 35)
ArsR (TF)	As^3+^^4^	sfGFP (fluorescence)	*in vitro*^5^	0.125 μM^6^	(87)
AtoSC (TCS)	acetoacetate (C_4_)	GFP (fluorescence)	*E. coli*	0.001–10 mM	(28)
BmoR (TF)	various SMCAHs (C_3_–C_6_)	GFP (fluorescence)	*E. coli*	0.1–40 mM^3^	(33)
CadC (TF)^7^	Pb^2+^, Cd^2+^	Luc (bioluminescence)^8^	*E. coli*	0.01–10 μM	(53)
CadC (TF)^7^	Cd^2+^^9^	sfGFP (fluorescence)	*in vitro*^5^	0.5 μM^6^	(87)
CadR (TF)^10^	Cd^2+^^10^	GFP (fluorescence)	*E. coli*	3 nM^6^,^10^	(54)
CadR (TF)^11^	Cd^2+^, Hg^2+^	multiple read-outs^11^	*E. coli*	0.625–2.5 μM^12^	(56)
ChnR (TF)	various lactams	mCherry (fluorescence)	*E. coli*	1–100 mM	(62)
Co/Ni apt (RWS)	Co^2+^, Ni^2+^	mCherry (fluorescence)	*E. coli*	13	(58)
DmpR (TF)	*o*-cresol (C_7_)	mCherry (fluorescence)	*E. coli*	0.1–5 μM	(43)
FerC (TF)	feruloyl-CoA^14^	sfGFP (fluorescence)	*E. coli*	0.001–1 mM	(73)
FrmR (TF)	formaldehyde (C_1_)	multiple read-outs^15^	*E. coli*	1–25 μM	(21)
F^–^ apt (RWS)^16^	F^–^	sfGFP (fluorescence)	*P. putida*	2.5–10 mM^17^	(49)
Haa1-BM3R1 (TF)^18^	organic acids (C_1_–C_6_)	mCherry (fluorescence)	*S. cerevisiae*	10–60 mM^18^	(19)
hsFRET (probe)^19^	H_2_S	cpsGFP-EBFP2 (FRET; fluorescence)	*in vitro*/*in vivo*^20^	10–100 μM^19^	(45)
HyPer7 (TF)^21^	H_2_O_2_	cpGFP (fluorescence)	*in vitro*/*S. cerevisiae*	20–100 μM	(39)
Hypocrates (TF)^22^	(pseudo)hypohalous acids	cpYFP (fluorescence)	*in vitro*/*in vivo*^20^	0.1–0.33 μM^6^	(41)
Leu3p (TF)	2-IPM (C_7_)^23^	GFP (fluorescence)	*S. cerevisiae*	10–80 μM	(32)
LuxAB (enzyme)	various aldehydes	LuxAB (bioluminescence)	*E. coli* or *A. baylyi*	0.1 mM^6^,^24^	(36, 37, and 75)
MerR (TF)	Hg^2+^	sfGFP (fluorescence)	*in vitro*^5^	0.011 μM^6^	(87)
MGapt (RWS)	malachite green	GFP (fluorescence)	*in vitro*^5^	0.0375–3.0 μM^25^	(88)
OxyR (TF)	hydrogen peroxide (H_2_O_2_)	multiple read-outs^26^	*E. coli*	5–100 μM^17^	(43)
PbrR (TF)^27^	Pb^2+^	GFP (fluorescence)	*E. coli*	0.001–100 μM^28^	(52)
PbrR (TF)^27^	Pb^2+^	Luc (bioluminescence)^8^	*E. coli*	1–100 μM	(53)
PcaV (TF)	protocatechuic acid (C_7_)	sfGFP (fluorescence)	*E. coli*	0.1–1000 μM^29^	(73)
pnGFP-Ultra (probe)	peroxynitrite (ONO_2_^–^)	cpsGFP (fluorescence)	*in vitro*/*in vivo*^20^	122 nM^6^	(40)
PREmR34 (RWS)	protons (pH sensor)	mCherry (fluorescence)	*E. coli*	pH = 5.0–8.0	(72)
PyronicSF (TF)^30^	pyruvate (C_3_)	cpGFP (fluorescence)	*in vitro*/*in vivo*^20^	30	(22)
Tsa2-GFP (enzyme)^31^	H_2_O_2_	roGFP (fluorescence)	*in vitro*/*S. cerevisiae*	1–100 μM^31^	(39 and 44)
War1 (TF)	various SMCFAs (C_3_–C_7_)	GFP (fluorescence)	*S. cerevisiae*	0.5–25 mM^32^	(29 and 30)

1 GFP output at 40 mM *n*-alkane concentration.

2 Linear range for 1-pentanol.

3 Linear range for 1-butanol.

4 Response also to As^5+^, Cd^2+^, Hg^2+^, and Pb^2+^ ions.

5 TF and sfGFP transcribed/translated by cell-free expression (CFE) systems.

6 Lower limit of detection (LOD).

7 *Staphylococcus aureus* (*S. aureus*) CadC.

8 Luc, firefly luciferase.

9 CFE also induced by Pb^2+^ and Hg^2+^.

10 *Halomonas caseinilytica* CadR; biosensor sensitivity increased by ZitB transporter, minimizing Zn^2+^ interference.

11 *Pseudomonas putida* CadR; reporters: fluorescent proteins (GFP or mCherry) and LacZ (β-galactosidase).

12 For Cd^2+^; induction by Hg^2+^ >2.5 μM.

13 Detection range: Co^2+^ <1 mM; Ni^2+^ <2 mM.

14 Feruloyl-CoA produced from ferulic acid by FerA.

15 GFP (fluorescence) or LuxAB (bioluminescence).

16 Apt, aptamer.

17 Linear range; for maximal nonlinear response, see reference.

18 BM3R1 DBD fused to the TF Haa1; operational range for acetic acid (C_2_).

19 CpsGFP-EBFP2 fusion probe; linear change in FRET ratio *in vitro* with hsFRET (1 μM) and tested H_2_S concentrations.

20 For mammalian cells and/or animal model, see reference.

21 OxyR-GFP fusion.

22 NemR-YFP fusion.

23 2-IPM detected as byproduct or precursor, indicating isobutanol (C_4_) or isopentanol (C_5_) production, respectively.

24 Higher aldehyde concentrations regularly cytotoxic.^36,75^

25 Based linear calibration with purified MGapt-GFP transcript.^88^

26 mCherry or GFP.

27 *Cupriavidus metallidurans* PbrR.

28 LOD of most sensitive biosensor variant (S23); sensitivity increased by LuxR as signal amplifier.

29 Sensitivity enhanced by protocatechuic acid-specific transporter PcaK.

30 PdhR-GFP fusion; changes in pyruvate concentrations as low as 10 μM detected (e.g., mean mitochondrial pyruvate concentrations = 25 μM).

31 Thiol peroxidase Tsa2 fused to H_2_O_2_-sensitive roGFP;^44^ detection range according to Pak and co-workers.^39^

32 Based on isovaleric acid (C_5_); concentrations >25 mM not tested.

In the last two decades, the utilization of biosensors has advanced beyond their initial use as analytical tools for the high-throughput (HT) detection and quantification of natural metabolites and xenobiotics. Today, biosensor systems complement well-established but low-throughput chromatographical methods and have facilitated the directed evolution of proteins, the engineering of (natural) metabolic pathways, and their dynamic control. This resulted in the construction of microbial cell factories for the detection of various small molecules and the efficient manufacturing of value-added platform chemicals, of which selected examples, focused on biosensing, will be featured in the following.^10,13,14^

## Biosensors for Carbon Fixation Products and Derivatives

Currently, the fixation of CO_2_ through the Calvin-Benson-Bassham cycle mainly by autotrophs (e.g., plants, algae, cyanobacteria) and a few recently discovered natural carbon-fixation pathways in bacteria and archaea cannot balance the excessive anthropogenic CO_2_ emission.^5^ Very recently, Gleizer et al. constructed and evolved *Escherichia coli* (*E. coli*) to produce all its biomass carbon from CO_2_*via* the Calvin-Benson-Bassham cycle, enabling autotrophic growth of an otherwise heterotrophic bacterium.^15^ Complementary, significant efforts have been made not only to transplant and improve natural carbon fixation but also to design artificial assimilation routes.^1,5^ While many natural and *de novo* CO_2_ fixation pathways depend on the same energy carriers such as ATP and (phosphorylated) nicotinamide cofactors, fixation products and pathway intermediates can be fairly distinct.^5,6^ Besides the challenging implementation of synthetic pathways in microorganisms, biosensors might not have become available to sense associated small molecules.^1,5,10^ This is also true for the CETCH (crotonyl-CoA/ethylmalonyl-CoA/hydroxybutyryl-CoA) cycle, assembled by Schwander and co-workers, for the fixation of two molecules CO_2_*in vitro*.^16^ Furthermore, many immediate CO_2_ fixation products are quickly metabolized and do not accumulate in living (micro)organisms, calling for qualitative and quantitative measurement tools that are highly sensitive to evaluate engineering efforts.

Biosensors have been developed and employed to match these requirements but have yet to be implemented to accelerate the optimization of (artificial) CO_2_ assimilation pathways and to complement (indirect) performance assessments such as growth assays.^10,17^ Hence, biosensor systems that have been developed for other purposes but are suitable to detect carbon fixation products and related molecules will be highlighted in the following.^5,6^

One industrially important CO_2_ fixation product is formate (C_1_; 46.03 g mol^–1^), for example. Since it can be produced (enzymatically) from multiple (renewable) sources and further converted into platform chemicals like formaldehyde and pyruvate, formate is of interest to different industries.^5,18^ Mormino et al. constructed a biosensor, discussed for biosensing formate, by fusing the *Saccharomyces cerevisiae* (*S. cerevisiae*) TF Haa1 and the DBD of BM3R1 from *Bacillus megaterium*. Whereas promotors containing Haa1 binding sites did not drive reporter gene expression, the production of mCherry, a red fluorescent protein (RFP), could be tuned by synthetic promoters containing different numbers of BM3R1 binding sites in the presence of various C_1_–C_6_ acids, including formate, acetate (C_2_; 60.05 g mol^–1^), propionate (C_3_; 74.08 g mol^–1^), and lactate (C_3_; 90.08 g mol^–1^).^19^

Similarly, formaldehyde (C_1_; 30.03 g mol^–1^) is a versatile chemical building block. Lu et al. implemented an artificial fixation route for formaldehyde operating in *E. coli*.^20^ Their synthetic acetyl-coenzyme A (SACA) cycle condenses two molecules of formaldehyde into glycolaldehyde. The latter is further converted into acetyl-coenzyme A (CoA), which is a central metabolite for the biosynthesis of numerous products as discussed below. Due to its high reactivity, formaldehyde is considered an environmental toxin and must be carefully monitored.^2,21^ Accordingly, Woolston et al. developed a formaldehyde biosensor based on the FrmR repressor protein and the cognate *P*_*frm*_ promoter sequence of *E. coli*. The native TF binding site was rationally engineered and allowed detection as low as 1 μM, utilizing the luciferase LuxAB from *Photorhabdus luminescens* or GFP as readouts. Ultimately, the biosensor system was used to characterize methanol dehydrogenase variants *in vivo*.^21^

Arce-Molina et al. developed a highly responsive sensor for pyruvate (C_3_; 88.06 g mol^–1^), another carbon fixation product.^22^ Pyruvate is a central molecule in carbon metabolism and a precursor for acetyl-CoA, which fuels numerous vital metabolic pathways.^5,20^ The biosensor PyronicSF comprises the complete sequence of the bacterial TF PdhR, which is linked to a circularly permuted version of green fluorescent protein (cpGFP).^22^ CpGFPs or other fluorescent proteins contain engineered termini that are fused to sensing domains. The conformational rearrangement of the latter modulates the fluorescent intensity.^23^ Exposure of the PdhR-GFP sensor to pyruvate causes an increase in fluorescence when excited by blue light. Importantly, their biosensor remained insensitive to acetate, lactate, and other structurally related C_2_–C_6_ acids.

As introduced above, acetyl-CoA is ubiquitous in living organisms and a key intermediate in the biosynthesis of (branched) short- and medium-chain fatty acids (SMCFAs), the corresponding alcohols (SMCAHs), and *n*-alkanes, among other compounds. Particularly, SMCFAs and SMCAHs are considered valuable carbon and energy sources and have applications as drop-in biofuels.^24,25^ Consequently, the biosensor-based engineering of microbial cells to sustainably produce these molecules is of great interest.

Rutter et al. utilized a TCS from *E. coli* to detect acetoacetate (C_4_; 102.09 g mol^–1^), which can be directly used as a carbon source and plays a crucial role in the synthesis of SMCFAs and poly-(*R*)-3-hydroxybutyrate, for example.^26,27^ The whole-cell biosensor consists of the AtoS histidine kinase, which autophosphorylates in the presence of acetoacetate. Subsequently, the phosphate group is transferred to the AtoC response regulator, which triggers the expression of *gfp* from the native promoter *P*_*ato*_. On the basis of a model-driven sensitivity analysis, Rutter et al. generated a range of input/output responses by tuning the concentration of AtoS/AtoC (genomic versus plasmid-based expression) and *P*_*ato*_ (low- versus high-copy number vector), aiming at optimizing the genetic context.^28^ Since TCS are highly susceptible to contextual effects, their engineering and contextualization will play a crucial role in broader future applications as briefly discussed later.

To directly monitor the production of SMCFAs, Baumann et al. developed a whole-cell biosensor that is based on a multicopy yeast plasmid, encoding *gfp* as the reporter gene under transcriptional control of the PDR12 promoter (*P*_*PDR12*_) and the constitutively bound TF War1. Upon exposure to SMCFAs (C_3_–C_7_), War1 undergoes phosphorylation and conformational changes, initiating GFP expression in *S. cerevisiae*.^29^ Similarly, Miyake et al. utilized *P*_*PDR12*_/War1 and GFP to detect the branched SMCFAs isobutyric acid (C_4_; 88.11 g mol^–1^), 2-methylbutanoic acid and 3-methylbutanoic acid (both C_5_; 102.13 g mol^–1^), as well as unsaturated methacrylic acid (C_4_; 86.06 g mol^–1^), and interestingly, benzoic acid (C_7_; 122.12 g mol^–1^).^30^ The authors also altered biosensor performance (e.g., sensitivity and operational range) by varying the expression levels of the weak organic acid transporter PDR12 and suggested the implementation of War1 mutants, displaying different binding affinities of the LBD and the DBD for branched SMCFAs and cognate operator sequences, respectively.^31^

For the detection of the corresponding (branched-chain) SMCAHs, Zhang et al. developed a TF-based biosensor system in *S. cerevisiae*.^32^ The TF Leu3p binds 2-isopropyl malate (2-IPM, C_7_; 176.17 g mol^–1^) and controls the expression of GFP. Two distinct biosensor configurations enable the HT screening for the enhanced production of isobutanol (C_4_; 74.12 g mol^–1^) or isopentanol (C_5_; 88.15 g mol^–1^) by monitoring 2-IPM as a byproduct or as a precursor, respectively.

In contrast, Yu et al. directly monitored the production of isobutanol in *E. coli* through the alcohol-regulated TF BmoR and its cognate promoter *P*_*bmo*_, driving the expression of GFP. In the natural host *Thauera butanivorans*, BmoR regulates an alkane monooxygenase involved in the metabolization of *n*-alkanes. The TF exhibits high sensitivity to linear and branched-chain SMCAHs (C_4_–C_6_ and C_3_–C_5_, respectively).^33^ A complementary approach was utilized by Bahls et al., who engineered the LBD of the TF AlkS by error-prone polymerase chain reaction (epPCR), importantly, without influencing the DBD.^34^ Wildtype AlkS accepts short- and medium-chain *n*-alkanes and the corresponding SMCAHs, with 1-pentanol (C_5_; 88.15 g mol^–1^) being the shortest alcohol detectable. Mutant AlkS also accepted isopropanol (C_3_; 60.10 g mol^–1^), butanol and 2-butanol, as well as isopentanol. *E. coli* cells expressing AlkS mutants and GFP were enriched by fluorescence-activated cell sorting (FACS) after exposure to the new ligands.^34^ In another study, epPCR had been used to enhance the binding profile of AlkS to *n*-alkanes (C_5_–C_9_).^35^

In the context of the detection and manufacturing of biofuels in living cells, the previously introduced monooxygenase LuxAB from *Photorhabdus luminescens* has been implemented versatilely. Bayer et al. coupled different heterologous oxidoreductases and LuxAB to monitor the production of aldehydes, including hexanal (C_6_; 100.16 g mol^–1^) and benzaldehyde (C_7_; 106.12 g mol^–1^), from carboxylic acid or primary alcohol substrates in an engineered *E. coli* K-12 MG1655 strain, exhibiting reduced aldehyde reduction activity (*E. coli* RARE).^36^ The structural relation of the investigated substrates to SMCFAs and SMCAHs might qualify this enzyme-coupled biosensor system not only for the HT detection of the corresponding aldehydes but the optimized biobased production of biofuels. Lastly, Lehtinen et al. combined the acetogen *Acetobacterium woodii*, to produce acetate from CO_2_ and H_2_, and an engineered *Acinetobacter baylyi* ADP1 (*A. baylyi*) strain. Albeit low-yielding, the second strain converted the produced acetate into *n*-alkanes. This was monitored by a twin-layer biosensor system: the formation of intermediate aldehydes was followed by LuxAB. A cyanobacterial aldehyde-deformylating oxygenase yielded, besides formate, target *n*-alkanes, which were sensed by the TF AlkR and the cognate *P*_*alkM*_ promoter, regulating the expression of GFP.^37^

So far, examples of the implementation of genetically encoded biosensors focused on the assessment of strategies for the conversion of C_1_ compounds into industrially important precursors and diversified products including biofuels (C_2_–C_7_). As pointed out earlier, many of these organic compounds do not accumulate in high quantities naturally.^10^ The C_1_ molecules methanol, formaldehyde, and formate, as well as *n*-alkanes exhibit cellular toxicity even at low concentrations and their assimilation as well as their detoxification requires (micro)organisms with unique enzymatic activities.^1,4,18,21,36^ Similarly, inorganic molecules leaking into the environment, even accumulating as seen with HMs, due to anthropogenic actions can be toxic.^2,4,38^ Hence, the following section will feature biosensor systems for the detection of selected inorganic compounds.

## Biosensing of Inorganic Molecules and Contaminants

Physiologically, many inorganic compounds such as metals, reactive oxygen and nitrogen species (ROS and RNS, respectively), or hypohalous acids are involved in important cellular processes.^39−41^ Consequently, not only the detection of inorganic pollutants is crucial; robust and highly sensitive biosensor systems enable the monitoring of biological functions and signaling events at single-cell resolution.

Hydrogen peroxide (H_2_O_2_; 34.02 g mol^–1^) is a ROS and a key intermediate of the aerobic metabolism. Different sensitive biosensors for the detection of H_2_O_2_, referred to as HyPer variants, have been developed and are based on circularly permuted yellow fluorescent protein (cpYFP) or cpGFP integrated into the regulatory domain of the *E. coli* TF OxyR, an H_2_O_2_-sensing regulator.^39^ A cysteine residue (Cys199) in OxyR is sensitive to oxidation by H_2_O_2_, but not other oxidants, resulting in a disulfide bond formation and a subsequent conformation change.^42^ Initially, 11 OxyR regulatory domains from different bacterial species were tested for the integration of cpYFP with varying insertion positions to optimize folding of the fluorescence domain. The most sensitive construct, using OxyR from *Neisseria meningitidis*, was further enhanced by protein engineering, using a combination of rational and random mutations. This optimized biosensor allowed *in vitro* detection of H_2_O_2_ concentrations in the lower micromolar range, while demonstrating high pH stability at physiological relevant conditions, an advantage to previous HyPer variants.^39^

Kardashliev et al. employed OxyR coupled to fluorescent reporters – GFP or RFP – to monitor H_2_O_2_ as a byproduct of the oxidation of glycerol to glyceraldehyde (C_3_; 90.08 g mol^–1^) and of toluene to *o*-cresol (C_7_; 108.14 g mol^–1^) by different recombinant oxidoreductases in *E. coli*. Additionally, *o*-cresol formation was followed by a second genetically encoded sensor, the phenol-sensitive transcriptional activator DmpR, which drives the expression of an orthogonal fluorescent reporter gene.^43^

A third biosensor for H_2_O_2_ utilizes the thiol peroxidase Tsa2 from *S. cerevisiae* genetically linked to a redox-sensitive GFP (roGFP). In this redox relay system, Tsa2 catalyzes the transfer of oxidizing equivalents from H_2_O_2_ to roGFP. Under endogenous conditions, the biosensor is about 50% oxidized, which allows measuring the increase and decrease of H_2_O_2_ in living cells with both high sensitivity and selectivity.^44^

Like ROS, RNS are associated with signal transduction and stress responses in mammalian cells. Hence, Chen et al. advanced an earlier biosensor design to develop a highly sensitive fluorescence probe for peroxynitrite (ONO_2_^–^; 62.01 g mol^–1^), using a circularly permuted superfolder GFP (cpsGFP) with the noncanonical amino acid *p*-boronophenylalanine incorporated into the chromophore.^40^ Several rounds of directed evolution yielded a fluorescence biosensor with a 110-fold turn-on response and high selectivity for peroxynitrite over other ROS/RNS. Peroxynitrite concentrations as low as 122 nM could be detected *in vitro* and *in vivo*.

(Pseudo)hypohalous acids, including hypochlorous acid (HOCl; 52.46 g mol^–1^) and hypobromous acid (HOBr; 96.91 g mol^–1^), are part of the immune response system that provides an effective defense mechanism against bacteria, fungi, and larger parasites. Kostyuk et al. developed a fluorescence biosensor consisting of cpYFP integrated into the transcriptional repressor NemR from *E. coli*, which senses hypochlorite anions.^41^ To optimize the fluorescence response, 16 different constructs with varying linker regions were designed and tested. The best construct, named Hypocrates, showed a 1.6-fold turn-on response and allowed the detection of HOCl, HOBr, *N*-chlorotaurine, and hypothiocyanous acid with a lower detection limit of 100–330 nM (for NaOCl and NaOBr). Hypocrates enabled measuring (pseudo)hypohalous acid derivatives in different mammalian cell lines and in a zebrafish model.

Recently, Youssef et al. developed a fluorescent biosensor for H_2_S (34.1 g mol^–1^), a toxic gas and an important biological signaling molecule. Detection is based on the incorporation of the noncanonical amino acid *p*-azidophenylalanine in an engineered GFP-blue fluorescent protein (BFP) fusion.^45^ First, cpsGFP was fused to the BFP EBFP2 by a short, flexible peptide linker and resulted in a Förster resonance energy transfer (FRET) pair. This chimeric fusion was then modified by genetic code expansion to incorporate *p*-azidophenylalanine into the chromophore of cpsGFP (Tyr154). The construct was improved through several rounds of directed evolution for increased FRET. This yielded the optimized mutant hsFRET, in which the azido group in *p*-azidophenylalanine is reduced to an amino group by H_2_S, resulting in enhanced FRET from EBFP2; hsFRET allowed the selective detection of H_2_S as low as 10 μM *in vitro*. Importantly, experiments in mammalian cells demonstrated that other redox-active molecules caused no signal response by hsFRET. Noteworthy, other fluorescence proteins incorporating noncanonical amino acids have been reported as well.^46,47^

Most of the TF-based biosensors detecting the inorganic molecules described above operated in eukaryotic organisms and subcellular compartments (e.g., mitochondria). Whereas their transplantation into microbial host cells remains to be demonstrated, the detection of HM ions, for example, was realized in different *E. coli* strains.

Many (divalent) ions fulfill essential cellular functions. While free intracellular levels of magnesium ions can reach 5 mM, the same concentration of divalent ions like nickel (Ni^2+^) or cobalt (Co^2+^) can inhibit growth; the F^–^ anion is toxic at elevated concentrations.^48,49^ HMs such as lead (Pb), cadmium (Cd), and mercury (Hg) exert toxic effects on living organisms at very low concentrations and are recalcitrant to degradation. Some of these HMs can be absorbed by plants and, thus, can potentially accumulate in the food chain.^38^ Pb and Hg have toxic effects on the nervous, digestive and immune systems, lungs, and kidneys. Similarly, exposure to low levels of Cd over time, particularly in tobacco smoke, may cause kidney disease. Cd is also considered a cancer-causing agent.^50,51^ Consequently, the leakage of HMs from industrial and agricultural activities into the environment is of concern, and biosensors for their sensitive, fast, and low-cost monitoring are desired.

Jia et al. improved a microbial whole-cell biosensor for Pb (207.2 g mol^–1^).^52^ In *Cupriavidus metallidurans* CH34, the TF PbrR regulates a gene operon conferring resistance to enhanced levels of Pb^2+^ ions from the divergent promoter *P*_*pbr*_. Synthetic PbrR-based gene circuits were constructed featuring different genetic architectures tuning the expression of *gfp* as the reporter gene. Furthermore, a positive-feedback amplifier employing a variant of the quorum-sensing regulator LuxR was introduced to improve sensitivity to Pb^2+^ ions and increase the fluorescent output signal. In genetic configurations featuring positive feedback loops, the divergent promoter *P*_*pbr*_ also controlled the expression of *luxR*, which binds to its cognate promoter *P*_*luxI*_. The latter drives the expression of the *gfp* reporter gene and an additional copy of *luxR* amplifier, which increased the output signal up to 1.9-times compared to biosensors without positive feedback.^52^ Biosensors operating in *E. coli* DH5α were able to detect Pb^2+^ at a concentration as low as 0.01 μM; the standard drinking water quality requirement is <0.05 μM.^51^

Similarly, Nourmohammadi et al. compared two Pb biosensors with luciferase reporter gene expression either controlled by PbrR/*P*_*pbr*_ from *Cupriavidus metallidurans* CH34 or CadC/*P*_*cad*_ from *Staphylococcus aureus* (*S. aureus*) in *E. coli* DH5α.^53^ Whereas the PbrR-based construct detected Pb concentrations of 1–100 μM without interference by other metal ions, including Cd^2+^, Ni^2+^, and zinc (Zn^2+^), the CadC-based biosensor could detect Pb^2+^ concentrations between 10 nM and 10 μM.

He et al. genetically tuned the metal transport system of *E. coli* to enrich intracellular Cd^2+^ ions, while reducing the concentration of other interfering metal species *in vivo*. The TFs CadR from *Pseudomonas aeruginosa* and *Pseudomonas putida* (*P. putida*) were coupled to GFP as the readout. Although these biosensors were able to detect Cd (112.41 g mol^–1^) at relatively low concentrations of 1 μM, an unspecific response to Hg^2+^, Pb^2+^, and Zn^2+^ ions, for example, was observed. After an extensive sequence similarity search, 14 more Cd-sensing TFs were selected and tested. The resulting Cd biosensor featured CadR from *Halomonas caseinilytica* JCM 14802 and the *E. coli* metal transporter ZitB. The system was highly specific for Cd^2+^ and showed a detection limit of 3 nM.^54^ ZitB preferentially ejects Zn^2+^ ions, thereby, minimizing interference.^55^

The natural *cad* operon of *P. putida* is controlled by the repressor CadR, binding to its cognate divergent promoter (*P*_*cad*_). In the presence of Cd, the dissociation of CadR increases the transcription of its own gene and a Cd efflux pump. Guo et al. redesigned the operon, generating a novel cell-based bioadsorption device for Cd^2+^ ions as well as different multiple-signal biosensor modules for their detection *in vivo*.^56^ Bioadsorption was facilitated by the Cd-binding domain (CdBD) from CadR fused to the C-terminus of the surface display protein Lpp-OmpA. The chimeric protein Lpp-OmpA-CdBD can bind Cd on the cellular surface. Although it remained unclear whether enriched Cd on the surface of whole-cell biosensors contributed to elevated intracellular concentrations of Cd, and consequently, an increased GFP output, the surface adsorption of Cd^2+^ ions was up to 8.8-fold higher than with *E. coli* TOP10 cells lacking CdBD display. Biosensor constructs employed the reporters mCherry, GFP, and LacZ (encoding a β-galactosidase) in different combinations under the control of CadR/*P*_*cad*_ to generate multiple readouts. The lower limit of detection for Cd^2+^ ions was 0.1 μM; strong output signals were also observed in the presence of Hg^2+^ ions.

Regarding the detection of the biologically essential metal ions Ni^2+^ (58.69 g mol^–1^) and Co^2+^ (58.93 g mol^–1^), Wang et al. employed a previously described Co/Ni-specific RSW by Furukawa and co-workers.^57^ Their sensor responds to increased intracellular ion levels, regulating the expression of mCherry fluorescent protein in *E. coli* DH5α. Subsequently, the RSW was used to assess the influence of gene deletions, Δ*rcnA*, Δ*rcnB*, Δ*rcnR*, Δ*nikA*, and Δ*nikR*, related to Co/Ni homeostasis and detoxification in *E. coli* K12 strains. Lower limits of reporter gene induction were 50 μM and ≥1 mM for Co^2+^ and Ni^2+^, respectively.^58^

Lastly, Calero et al. constructed synthetic RSWs responsive to F^–^ (18.99 g mol^–1^). For biosensing, different (artificial) promoters controlled the production of superfolder GFP (sfGFP) in *P. putida*.^49^ Discernable sfGFP outputs above background levels could be detected at 2.5 mM sodium fluoride (NaF) and increased 200-fold at 15 mM; deletion of the *crcB* gene (encoding a F^–^ exporter) inhibited growth at NaF concentrations ≥0.5 mM. Subsequently, Calero et al. coupled the translation of an orthogonal T7 RNA polymerase (RNAP) to the RWS. The T7 RNAP facilitates the T7 promoter (P_*T7*_)-controlled expression of various fluorinases and a purine nucleotide phosphorylase to synthesize fluorosugars and fluoronucleotides *in vivo*.

## Identification of Novel Biosensor Systems and Contextualization Strategies *In Vivo*

Biosensing and signal transduction in response to chemicals in the environment are common features in all living (micro)organisms and essential to regulate cellular functions including growth and survival.^10,12^ Key players are the naturally evolved sensory devices including TFs, RSWs, and TCS (Figure 1A–C). In particular TF- and RSW-based biosensor systems have been utilized, engineered, and contextualized to efficiently detect (non-natural) compounds as highlighted above. To facilitate the detection of small molecules for which no natural or engineered sensory part has become available yet, tools for the identification of novel biosensors as well as the adaptation of biosensor performance in the cellular context are vital.

In recent years, the number of characterized RSWs as sensing tools has not increased as rapidly compared to TFs although RSWs are predicted to be broadly distributed gene regulatory elements in bacteria, fungi, and plants but are conspicuously absent in animals.^10,59^ This is also deducible from the overrepresentation of TF-based biosensor systems in this focused review (Table 1). Similarly, TCS are a vast group of proteins, predicted to be the biggest group of signal transduction pathways in biology. Due to their only recent introduction as biosensor devices, conserved regulatory motifs and variability in TCS across (microbial) genomes, determining signaling mechanisms, their integration in gene regulatory networks, and ligand-binding scopes are still uncharacterized. Additionally, first engineering approaches indicate that TCS are highly susceptible to contextual effects, impeding their broader application.^12,60,61^

So far, TFs have been successfully identified by combined transcriptome and proteome analyses after exposure of (microbial) cells to the desired small molecule as well as the computer-assisted mining of databases. Furthermore, (prokaryotic) TFs can be responsive to different but structurally related compounds (e.g., War1 for SMCFAs,^29,30^ BmoR,^33^ and AlkS^34,35^ for SMCAHs, or CadC for Cd^2+^, Pb^2+^, and other HM ions^53^). This ligand binding promiscuity facilitated the random mutagenesis by epPCR and the rational engineering of various LBDs and DBDs by well-established protein engineering techniques.^10,17,31,32,34,35^ A complementary approach for the identification of new biosensors was employed by Zhang et al., who scouted chemicals with molecular shapes similar to the target small molecule. Bioinformatic-assisted gene cluster analysis identified the TF ChnR and the cognate promoter P_*b*_ as a biosensor for the non-natural γ-butyrolactam (2-pyrrolidone; 85.11 g mol^–1^), δ-valerolactam (2-piperidone; 99.13 g mol^–1^), and ε-caprolactam (azepan-2-one; 113.16 g mol^–1^). Detection of these platform chemicals was based on mCherry with a linear range of induction at 1–100 mM.^62^

Curated databases for prokaryotic and eukaryotic TFs such as PRODORIC (https://www.prodoric.de)^63^ and JASPAR (https://jaspar.genereg.net),^64^ respectively, include ligand binding profiles or the type of regulation (e.g., transcriptional activation and repression) together with cognate gene regulatory elements, which can assist initial biosensor designs. Similarly, databases and prediction tools such as Rfam (http://rfam.org)^65^ and the Riboswitch Scanner (http://service.iiserkol.ac.in/~riboscan/),^66^ respectively, are valuable sources of prokaryotic RSWs, providing information about their RNA aptamer sequences, conserved secondary structures, and known ligands. Despite the simple architecture of RSWs, the limited understanding of ligand-induced structural changes and the strong genetic contextual dependency impair their rational design.^10,49,67^ Hence, engineering examples exist but are scarce.

The systematic evolution of ligands by exponential enrichment (SELEX) has been successfully employed, during which a library of oligonucleotides specifically binding the target ligand can be enriched *in vitro*.^68^ During classical SELEX, only RNA aptamers with high-affinity binding were selected, while accompanying essential conformational changes had been neglected. Recently, this was addressed by various groups through RNA Capture-SELEX protocols, for example.^69−71^ This strategy involves the insertion of a small defined motif within the randomized region of RNA aptamer libraries. This “docking sequence” is hybridized through base pairing with a complementary oligonucleotide containing a linker molecule and biotin, which is anchored on streptavidin-coated magnetic beads. Only aptamers undergoing a structural rearrangement upon addition of the target ligand will be eluted from the beads.

**Figure 2:**
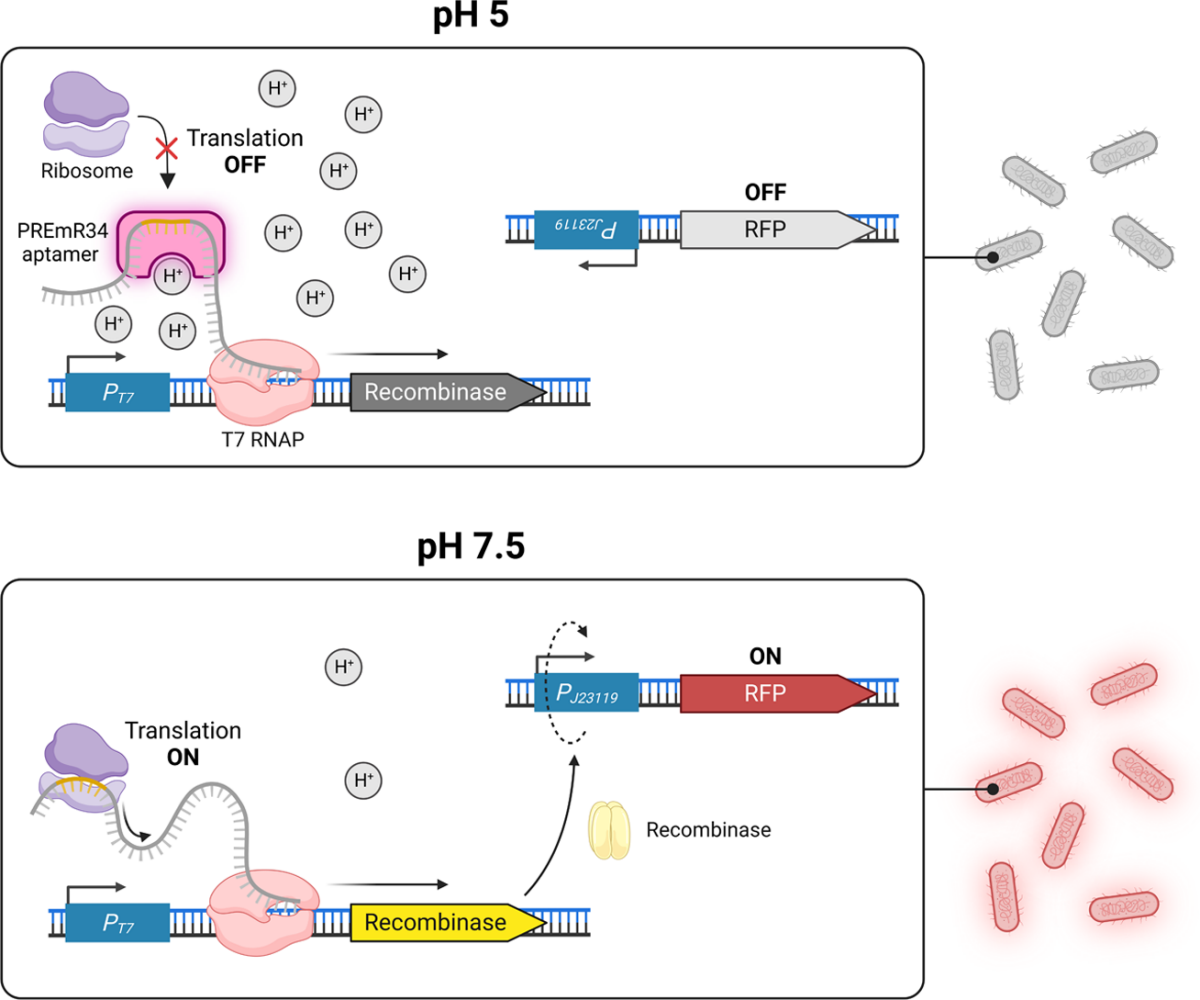
Contextualization of the pH-responsive RWS-based biosensor PREmR34. Transcripts are produced by a (mutant) T7 RNAP in *E. coli*. At low external pH, the strong RBS encoded within the aptamer (region highlighted in pink) is inaccessible for the ribosome (OFF state; top). At elevated pH, translation of a recombinase occurred, switching the orientation of the *P*_*J23119*_ promoter. Subsequent transcription and translation yield RFP as the readable output (ON state, bottom). Multilevel optimization approaches greatly increased the dynamic range of the target RWS.^72^ Figure created with BioRender.com.

One prominent engineering example by Pham et al. features a set of pH-responsive RSWs, enabling the production of RFP in response to changing environmental pH conditions (Figure 2).^72^ At an extracellular pH of 6.8, the wild-type RWS adopts a structure with a (weak) ribosome binding site (RBS), inaccessible for the ribosome, yielding translationally inactive transcripts (OFF state). Under an extracellular pH of 8.0, conformational changes lead to the formation of translationally active transcripts with an accessible RBS (ON state; Figure 2, bottom). For optimized biosensor performance, Pham et al. had to employ multiple strategies. First, the weak natural RBS of the RWS was replaced by a strong artificial one. Alleviating the leakiness of the pH-responsive sensor devices was equally important. Whereas tuning the *P*_*T7*_ strength was less effective, the implementation of a T7 RNAP with mutations in the active site (F644A and Q649S) to modulate transcription speed was crucial. Orthogonal expression of the T7 RNAP mutant was controlled by the inducer l-arabinose and carefully adjusted. Lastly, the engineered pH-sensitive RWS (PREmR34) downstream of *P*_*T7*_ regulated the expression of a recombinase. At a low extracellular pH of 5.0, the recombinase is repressed, maintaining *E. coli* cells in their OFF state (no fluorescence; Figure 2, top). At elevated pH, PREmR34 allows recombinase expression. This triggers the switching of the physical direction constitutive promoter J23119 (*P*_*J23119*_), embedded within the recombinase recognition sites (*attB* and *attP*). An insulator sequence upstream of the RFP-coding region was inserted to minimize potential context effects of the long 5′-untranslated region (UTR) introduced by the *attB* and *attP* flanking sites. Transcription of *P*_*J23119*_ and subsequent translation led to high red fluorescence (ON state; Figure 2, bottom). In the final genetic circuit, PREmR34 increased the OFF/ON fluorescence output up to 31-fold with a broadened dynamic range (pH 5.0–8.0). To illustrate the practicality of the engineered RWS system, pH sensing was linked to an artificial error-prone genome replication machinery, representing a novel RSW-based directed evolution (RiDE) protocol. Pham et al. employed RiDE, assisted by automated sampling, HT flow cytometry, and FACS, to isolate *E. coli* mutants tolerant to increased amounts of industrially important organic acids (C_2_–C_5_). This is a requisite for *E. coli* strains to be employed as production hosts.^72^

The development of PREmR34 (Figure 2) and selected examples featuring TFs above highlight the importance of contextuality for biosensors to operate optimally *in vivo*. Biosensor performance is not only affected by the attributes of the sensory part including the ligand specificity of TFs and RSWs. Sensitivity and operational range is defined as the concentration of the target small molecule (i.e., input signal) required for the biosensor to provide a significant change in the output signal (e.g., fluorescence) above host background.^10,11,13^

Obviously, adjusting biosensor expression is as important as tuning reporter gene levels and can be achieved by engineering the regulatory elements in the 5′ and 3′ UTR, including (natural and synthetic) promoters,^19,21,49^ transcriptional terminators, as well as the composition of RBS and adjacent nucleotides. This genetic contextualization is crucial to optimize the functionality of the biosensor.^9−11,72,73^ Additionally, the stoichiometry of sensory modules (TFs and RSWs) and binding targets (e.g., small molecules for TFs and RSWs or DNA binding sites for TFs) greatly influences sensitivity and the operational window.^10,19^

The necessity of iterative rounds permutating different combinations of genetic parts is laborious, time-consuming, and results are often nonintuitive.^10,11,73^ For example, the PbrR-based biosensor variant S23 showed the highest sensitivity for Pb^2+^ ions when production of the PbrR TF and the GFP reporter was controlled by the same (unidirectional) promoter.^52^

Berepiki et al. addressed this issue and used a design of experiments (DoE) methodology to systematically map combinations of genetic elements to greatly improve the performance of TF-based biosensor systems for protocatechuic acid (C_7_; 154.12 g mol^–1^) and ferulic acid (C_10_; 194.18 g mol^–1^), two catabolic breakdown products of lignin biomass. Noteworthy, Berepiki et al. could engineer the strong digital behavior of the investigated biosensor systems, characterized by high signal-to-noise ratios and a sigmoid dose–response curve, into a more analogue dose response. While the first allows one to confidently assign small molecule concentrations above a required threshold as desired in the initial screening of genetic libraries or the environmental monitoring of pollutants, analogue biosensor behavior (i.e., shallow or linear dose–response curve) is more appropriate to distinguish different variants or samples with similar activities, for example, as a result of protein engineering campaigns. Hence, the latter allows one to accurately distinguish subtle changes in analyte concentrations.^73^ In summary, the presented DoE approach promises to be a generalizable methodology to optimize other genetically encoded biosensor systems.

Furthermore, the cellular environment of host cells (i.e., host context) can strongly influence biosensor performance. Woolston et al. reduced the lower detection limit of formaldehyde in *E. coli* S1030 Δ*frmA* from 10 μM to 5 μM compared to the wildtype strain, in which FrmA is important for the detoxification of formaldehyde. However, variability in the GFP output signal was observed in the Δ*frmA* background.^21^ Furthermore, Bayer et al. showed that the use of *E. coli* RARE, exhibiting increased intracellular aldehyde persistence due to the knockout of endogenous alcohol dehydrogenases and aldo-keto reductases,^74^ improved the fold-increase in bioluminescence above background emitted by the luciferase LuxAB in the presence of various aldehydes.^36,75^ While the detection of terephthalic acid (TPA)-derived aldehydes such as 4-formylbenzoic acid and terephthalaldehyde (C_8_; 150.13 g mol^–1^ and 134.13 g mol^–1^, respectively) was feasible in different *E. coli* strains, the semiquantitative correlation between bioluminescence output and TPA concentration only succeeded in *E. coli* RARE.^75^ This enzyme-coupled biosensor system was one of the first to assess the activity of different (engineered) enzymes for the degradation of poly(ethylene terephthalate) and highlights the importance of host context for biosensor performance.

The knockout of genes otherwise responsible for the metabolization of target compounds contributed to increased intracellular concentrations, ultimately, increasing sensitivity. Similarly, Miyake et al. enhanced the intracellular concentration of SMCFAs by adjusting expression levels of PDR12, a transporter for organic acids. This strategy increased the sensitivity and the operational window of their SMCFA-responsive biosensor (*P*_*PDR12*_/War1 coupled to GFP).^30^ Complementary, export proteins can be used to reduce intracellular concentrations of potentially interfering chemical entities. One example from above featured the reduction of intracellular Zn^2+^ ions by the overexpression of the ZitB metal transporter to minimize interference with the CadR-based detection of Cd^2+^ ions in *E. coli*.^54^ Related knockout and knock-in strategies have also enabled the engineering of the central carbon metabolism in biotechnological hosts like *E. coli* and *S. cerevisiae*. Since the resulting strains can accumulate different carbon metabolites, including CO_2_ fixation products such as pyruvate (see above), they might provide suitable hosts to put related biosensor systems to the test.^10,76,77^

Lastly, the nature of reporter genes dictates their applicability and can strongly influence biosensor performance, particularly in the context of living cells. Commonly used reporter proteins include fluorescent proteins, (bacterial) luciferases, and metabolic enzymes such as LacZ (Table 1), and their unique advantages and disadvantages have been discussed earlier.^78−81^ Although dependent on the excitation by an external light source and potential interference with background fluorescence, one major advantage of autofluorescent proteins like GFP is that the generated amount of fluorescence is independent of exogenous substrates and usually stable over the monitoring time. Depending on the biosensor design, the fluorescent output can be correlated to the concentration of the target analyte, allowing quantification. Hence, variants of fluorescent proteins have become indispensable reporters in biosensing and HT applications including FACS.^10,34,72^

Bacterial luciferases are encoded by the *luxAB* genes and dependent on oxygen and reduced flavin mononucleotide cofactors.^81,82^ They emit bioluminescence in the presence of decanal (C_10_; 156.20 g mol^–1^) and other aldehyde substrates, either added directly or produced enzymatically from precursors.^36,37,75^ This allows the (near) real-time monitoring of aldehydes in living cells. Furthermore, bioluminescence can be autonomously produced if the complete *luxCDABE* operon is expressed. Coupled with a biosensor, such fully autonomous bioluminescent reporters can be employed remotely since aldehyde addition or *in situ* production are not required for expression.^78,81^ In general, luciferase enzymes, also including the well-known firefly luciferase, are good photoemitters in terms of quantum yield, and most cells are not luminescent, yielding highly sensitive detection tools with high signal-to-background ratios.^36,81,82^ However, the transient nature of bioluminescence signals might complicate quantification but examples exist.^53,75,78^

## Summary and Future Directions

The presented examples for TF- and RSW-based biosensors as well as enzyme-coupled systems and emerging TCS illustrate that biological parts are capable of “sensing the smallest”, compounds with low to average molecular masses and physicochemical properties that require time-consuming sample preparation and specialized instrumentation for their detection and quantification, rendering the analysis of these chemicals inflexible and costly. Contrarily, genetically encoded biosensors operating in whole cells are versatile, transferable, and cheap tools for the qualitative and (semi)quantitative analysis of small molecules.^10,12^ Advancements in the omics fields (e.g., transcriptomics, proteomics, and metabolomics) and bioinformatics have contributed to the discovery of TFs and RSWs, as well as their cognate natural genetic regulatory sequences. Improved DNA synthesis and recombineering technologies, together with HT screening strategies such as FACS, have enabled the permutation of natural and synthetic genetic parts to improve the performance of biosensor systems *in vivo*.^9−11,13^ The advent of synthetic biology has enabled the design, construction, and engineering of both sensory and transduction modules. Examples in this condensed review featured TFs with altered ligand specificities^10,31,32,34,35^ and chimeric TFs. The latter featured the combination of LBDs and DBDs from different regulatory proteins^19,56,83^ or covalent fusions of TFs to functional proteins including surface proteins^56^ and reporters.^22^ The engineering of reporters has yielded (circularly permuted) fluorescent proteins with customized properties, including varied excitation/emission wavelengths, for example, or split-systems being highly sensitive for target ligands.^10,12,23,41^ Recently, the incorporation of noncanonical amino acids further extended the repertoire of customized reporter proteins.^40,45−47^ The implementation of RSWs as biosensors is still underrepresented but protocols like Capture-SELEX^69−71^ and RiDE^72^ have been employed successfully to engineer RSWs to meet the required performance criteria.

In this regard, (genetic) contextualization remains a major challenge in biosensor development and (industrial) application. DoE methodologies, for example, already offer a valuable solution to greatly reduce the number of iterative rounds of permutating different genetic parts to customize biosensor performance.^10,73^ Furthermore, combinations of TFs and RSWs have been realized, yielding hybrid regulators in which the TF can compensate for the low dynamic range of the RSW and amplify the output signal.^84^ Current and future research will certainly target a more systematic and in-depth characterization of TCS, including the elucidation of ligand binding profiles, for applications in non-natural contexts and species including important biotechnological hosts.^12,60,61^ So far, the swapping of sensor kinase domains has been performed to engineer the detection scope toward the desired inputs.^12,85,86^ Nonetheless, success has been mere due to a limited understanding of the regulation and interactions of sensory domains and downstream response regulators. Although the latter contribute greatly to the variability and modularity in TCS families, they share high conservation in structure and regulation, suggesting that DBDs can be swapped to utilize alternative but well-characterized output promoters, driving the expression of reporter genes of choice.^12,84^

A major drawback of using living cells as sensing chasses is the inherent delay between input sensing and output generation, which is dictated by cell growth including protein synthesis and viability, for example.^10,13,87^ Some applications like assessing the quality of drinking water might require very short analysis times. For such purposes, cell-free expression (CFE) systems, harnessing the transcription/translation machinery of living cells to synthesize proteins *in vitro*, can be used.^10^ Beabout et al. developed three CFE biosensors to detect the HMs arsenic (As), Cd, and Hg.^87^ The TF/promoter pairs ArsR/*P*_*70a*_ (*E. coli*), CadC/*P*_*cad*_ (*S. aureus*), and MerR/*P*_*BBa_J23104*_ (*Shigella flexneri*) were coupled to sfGFP under the control of the cognate promoters *P*_*ars*_, *P*_*cad*_ (TF and reporter in operon configuration), and *P*_*merT*_, respectively. Noteworthy, successful transcription was determined by MGapt, a malachite green-sensitive RWS introduced previously to monitor RNA dynamics (Table 1).^88^ Engineering of these CFE biosensors led to the detection of 0.125 μM As^3+^, 0.5 μM Cd^2+^, and 0.011 μM Hg^2+^ ions in less than 30 min. Despite the low selectivity of the ArsR- and CadC-based biosensors, characterized by the detection of other HM ions like Pb^2+^ or Hg^2+^, output signals were obtained rapidly and meeting the recommendations by the World Health Organization for drinking water quality.^51,87^

In summary, the established biosensor systems are versatile analytical tools for the real-time monitoring of various metabolites and contaminants that are related to anthropogenic activities. Continuous advancements in bioinformatics and synthetic biology not only promise the discovery of novel sensor devices including their cognate gene regulatory parts but also customization strategies will certainly yield biosensor systems for the detection and quantification of non-natural small molecules including value-added platform chemicals and new-to-nature metabolites from synthetic CO_2_ fixation pathways, for example. Current biosensor applications showcased their use beyond mere sensing devices and included feedback control, the coordinated expression of multiple pathway genes, and the synchronization of cell populations, yielding microbial cell factories for the production of highly desired platform chemicals from nonfossil resource stocks.^10,13,14,37^ Furthermore, biosensors have been suggested to assess the degradation efficiency of plastic waste^75^ or to sequester highly toxic HM ions from the environment and adsorb them on the surface of bacterial cells.^56^ This points toward the integration of biosensors with new recycling and remediation strategies, tackling current and future environmental and socioeconomic challenges.

## Abbreviations

2-IPM: 2-isopropyl malate
ATP: adenosine triphosphate
*A. baylyi*: *Acinetobacter baylyi* ADP1
BFP: blue fluorescent protein
CdBD: cadmium-binding domain
CETCH: crotonyl-CoA/ethylmalonyl-CoA/hydroxybutyryl-CoA
CFE: cell-free expression
CoA: coenzyme A
cpGFP: circularly permuted green fluorescent protein
cpsGFP: circularly permuted superfolder GFP
cpYFP: circularly permuted yellow fluorescent protein
DBD: DNA-binding domain
DNA: deoxyribonucleic acid
DoE: design of experiments
epPCR: error-prone polymerase chain reaction
*E. coli*: *Escherichia coli*
FACS: fluorescence-activated cell sorting
FRET: Förster resonance energy transfer
GFP: green fluorescent protein
HM: heavy metal
HT: high-throughput
LBD: ligand-binding domain
LOD: lower limit of detection
*P*: promoter
RARE: reduced aldehyde reduction activity
RBS: ribosome binding site
RiDE: riboswitch-based directed evolution
RFP: red fluorescent protein
(m)RNA: (messenger) ribonucleic acid
RNAP: RNA polymerase
RNS: reactive nitrogen species
roGFP: redox-sensitive GFP
ROS: reactive oxygen species
RSW: riboswitch
SACA: synthetic acetyl-CoA
SELEX: systematic evolution of ligands by exponential enrichment
sfGFP: superfolder GFP
SMCAH: short-/medium-chain alcohol
SMCFA: short-/medium-chain fatty acid
*S. cerevisiae*: *Saccharomyces cerevisiae*
TCS: two-component systems
TF: transcription factor
TPA: terephthalic acid
UTR: untranslated region
